# Simulation-Based Mastery Learning Improves the Performance of Donning and Doffing of Personal Protective Equipment by Medical Students

**DOI:** 10.5811/westjem.2022.2.54748

**Published:** 2022-05-02

**Authors:** Danielle T. Miller, Nicholas Pokrajac, Jessica Ngo, Moises Gallegos, William Dixon, Kelly N. Roszczynialski, Kristen Ng, Nounou Taleghani, Michael A. Gisondi

**Affiliations:** * University of Colorado School of Medicine, Department of Emergency Medicine, Denver, Colorado; † Stanford University School of Medicine, Department of Emergency Medicine, Palo Alto, California

## Abstract

**Introduction:**

Medical students lack adequate training on how to correctly don and doff personal protective equipment (PPE). Simulation-based mastery learning (SBML) is an effective technique for procedural education. The aim of this study was to determine whether SBML improves proper PPE donning and doffing by medical students.

**Methods:**

This was a prospective, pre-test/post-test study of 155 medical students on demonstration of correct PPE use before and after a SBML intervention. Subjects completed standard hospital training by viewing a US Centers for Disease Control and Prevention training video on proper PPE use prior to the intervention. They then participated in a SBML training session that included baseline testing, deliberate practice with expert feedback, and post-testing until mastery was achieved. Students were assessed using a previously developed 21-item checklist on donning and doffing PPE with a minimum passing standard (MPS) of 21/21 items. We analyzed differences between pre-test and post-test scores using paired t-tests. Students at preclinical and clinical levels of training were compared with an independent t-test.

**Results:**

Two participants (1.3%) met the MPS on pre-test. Of the remaining 153 subjects who participated in the intervention, 151 (98.7%) reached mastery. Comparison of mean scores from pre-test to final post-test significantly improved from an average raw score of 12.55/21 (standard deviation [SD] = 2.86), to 21/21(SD = 0), t(150) = 36.3, P <0.001. There was no difference between pre-test scores of pre-clinical and clinical students.

**Conclusion:**

Simulation-based mastery learning improves medical student performance in PPE donning and doffing in a simulated environment. This approach standardizes PPE training for students in advance of clinical experiences.

## INTRODUCTION

Students are routinely exposed to blood, bodily fluids, and other potentially infectious agents during clinical rotations.^1^,^2^,^3^ To prevent dangerous occupational exposures, students must learn the proper technique for donning and doffing personal protective equipment (PPE).^4^ Recently, the coronavirus 2019 (COVID-19) pandemic placed further demands on medical schools and teaching hospitals to standardize PPE training for students ahead of their clinical experiences.

Despite these mandates, research shows that medical students receive inadequate training in PPE use, hand hygiene, and universal precautions.^5^–^8^ Moreover, PPE training programs commonly lack requirements for demonstration of technical proficiency.^9^ A study of PPE donning and doffing skills found that 92.5% of medical students had one or more lapses in procedural technique.^5^ Traditional teaching modalities such as computer-based modules, videos, lectures, and other PPE training approaches used by occupational health services have been found to not adequately prepare medical students for the clinical environment.^5^,^10^,^11^

Simulation-based mastery learning (SBML) is an instructional method that may address these training gaps. Mastery learning is an educational model that ensures all trainees meet a high, pre-defined standard for the learning.^12^ The SBML programs feature seven standard components including clearly defined learning objectives; a minimum passing standard (MPS) for proficiency; baseline skills testing using a standardized assessment; engagement in an educational activity with deliberate practice of a skill with expert feedback; post-testing to determine whether the MPS for mastery was achieved; continued practice until the mastery standard is reached; and advancement to the next educational activity only once the mastery standard is reached.^12^ Using SBML improves medical student performance of a wide range of procedural skills including peripheral intravenous catheter insertion, chest tube thoracostomy, laceration repair, chest compressions, and bag-valve-mask ventilation.^13^–^16^ To date, there have been no studies examining the use of SBML to teach PPE donning and doffing procedures to medical students.

In this study our aim was to determine whether SBML is an effective instructional technique for donning and doffing of PPE by medical students. The primary outcome was student performance of the procedure following a SBML intervention as assessed by a previously developed checklist. Secondary outcomes included a comparison of performance by students with and without previous clinical exposure.

## METHODS

### Study Design

This was a prospective, pre-test/post-test study of medical student subjects before and after a mastery learning educational intervention with a simulated clinical encounter. This study was deemed exempt by the institutional review board.

### Study Population and Setting

Eligible subjects included pre-clinical, second-year medical students enrolled in a Practice of Medicine (POM) course and clinical third- and fourth-year medical students completing a required emergency medicine (EM) clerkship. The study was conducted at a university-based teaching hospital (Stanford University School of Medicine) between July-December 2020. Students provided verbal informed consent prior to participation, and those who declined to participate in the research study still received the educational intervention.

**Population Health Research Capsule**

What do we already know about this issue?

*Medical students lack adequate training on how to correctly don and doff personal protective equipment (PPE).*

What was the research question?

*Is simulation-based mastery learning (SBML) an effective instructional technique for improving performance in donning and doffing of PPE by medical students?*

What was the major finding of the study?

*98.7% of students (151/153) achieved mastery in donning and doffing PPE after SBML.*

How does this improve population health?

*Medical schools should consider SBML when training students to don and doff PPE to better protect them prior to clinical experiences.*

### Study Protocol

We assessed subject performance of PPE donning and doffing techniques necessary for a clinical exposure to an airborne pathogen using a two-glove technique. Training occurred in a classroom space designed to simulate a medical examination room with a door and no anteroom. Students were asked to demonstrate donning and doffing of PPE for a patient under airborne precautions. We provided subjects standard PPE including a gown, two sets of non-sterile gloves, disposable goggles, and a simulated N-95 mask. Due to a national shortage of N95 face masks, we constructed simulated N95 masks using elastic bands stapled to an 8-ounce paper bowl, as described in a study by Pokrajac et al.^17^

The assessment tool was a previously published 21-item checklist of proper PPE use in a similar clinical training program.^17^ The checklist was created using PPE guidelines from the US Centers for Disease Control and Prevention (CDC) and Stanford Health Care Infection Control. The MPS was 21/21 items performed correctly, which was determined by a Mastery Angoff standard setting in the previous study.^17^ We similarly used this passing standard in our study, with one point awarded for items performed correctly and zero points for omitted or incorrectly performed items. Seven emergency medicine (EM) faculty members (four women, three men) completed asynchronous rater training using a 30-minute training video on administration of the mastery learning curriculum, expert demonstration of correct PPE use, checklist review, and mock assessments. To calibrate scoring, raters spent an additional 30 minutes using the checklist to score three standardized videos, and their responses were then compared. The seven EM faculty members also served as facilitators for the SBML session. All had completed a faculty-based SBML session on donning and doffing PPE, and all had experience facilitating simulation cases with medical students. Within the 30-minute training video, 10 minutes were dedicated to facilitator training specifically on the administration of the mastery learning curriculum.

Prior to the educational intervention, subjects completed standard hospital training on PPE donning and doffing by asynchronously viewing a CDC video on hand hygiene and PPE donning-and-doffing sequence. This video corresponded to the checklist items. Subjects had access to the video from three months to immediately before their session and were required to attest to viewing the video as part of a hospital-wide protocol. We scheduled two-hour sessions, with two facilitators who also served as raters and a maximum of 16 students. During the students’ scheduled PPE mastery learning session, they underwent baseline testing, deliberate practice with feedback, and post-testing. Baseline testing occurred after students asynchronously watched the CDC training video but before the mastery learning intervention. During the mastery learning session, all students had time for deliberate practice, including at least one demonstration of donning and doffing of PPE with targeted feedback, prior to post-testing. Deliberate practice occurred until the student felt comfortable with the procedure and that no more practice was required. Students who did not meet the MPS were provided additional deliberate practice until mastery was achieved.

### Data Collection

One trained faculty member rated students using the 21-item checklist during baseline and post-testing assessments. Prior to baseline testing, students completed a pre-survey that included demographic information and any additional PPE training beyond the required video viewing.

### Data Analysis

We performed statistical analysis using SPSS Statistics for Windows, version 26, (IBM Corp., Armonk, NY). Baseline test scores and final post-test scores were analyzed using two-tailed paired t-test. We analyzed within-group differences for POM, and EM subjects were analyzed using a two-sample t-test. We used central tendency metrics to summarize demographic and survey data.

## RESULTS

Of 168 eligible subjects, 155 completed baseline testing (Table), and 13 students did not due to schedule conflicts; 73 (62.9%) reported previous PPE training in addition to the hospital-required videos. Two students met the MPS at baseline assessment, leaving 153 students to participate in the mastery learning session. Of these, 151 subjects achieved mastery in post-testing, and two did not. Performance improved from a mean baseline testing score of 12.55/21 (standard deviation [SD] = 2.86), to a post-test mean score of 21/21(SD = 0), t(150) = 36.3, *P* <0.001) (Figure). There were no significant differences in mean baseline scores for pre-clinical students (POM, M = 12.64, SD = 3.15) and clinical students (EM, M = 12.80, SD = 2.76) t(153) = 0.30, *P* = 0.77). We calculated percent agreement on checklist rating of the three standardized videos for the seven raters (four women, three men), which was determined to be 96%.

## DISCUSSION

This study demonstrates the effectiveness of SBML to teach PPE donning and doffing technique to medical students with a standardized measure of skill proficiency in a simulated clinical environment. Student subjects in our study achieved the same mastery standards for the procedure as resident and attending physicians in a previously published SBML study.^17^ This suggests that SBML effectively trains individuals to properly use PPE, regardless of the degree of their prior clinical experience. This study adds to the growing body of literature supporting the use of mastery learning as the gold standard for teaching bedside skills and procedures in preparation for clinical practice.^13^–^16^

All but two subjects failed to meet the MPS at baseline testing for proper PPE use despite the usual medical school and hospital infection control training, ad-hoc clinical instruction on the wards, and mandatory viewing of a CDC video that mirrored our assessment checklist. This failure rate is similar to that in a 2016 study by John et al in which 98.9% of student subjects had one or more lapses in required PPE technique.^5^ Students in that study had similar rates of prior PPE training experiences to our study subjects and, importantly, none had been required to demonstrate procedural proficiency. These are very concerning findings, especially in light of the ongoing COVID-19 pandemic. It is imperative that medical schools improve PPE training and other infection control education to better protect their students for clinical experiences.

Two features of SBML likely contributed to achievement of a mastery standard in this study. First, SBML incorporates deliberate practice with expert feedback and no time limit for practice, leading to improved procedural competency.^13^–^15^ Standard hospital infection control training generally offers little if any skills practice, thus reducing its success in providing procedural competency. 9 Additionally, while most of participants in this study achieved mastery with deliberate practice and expert feedback, two participants in our study did not meet the MPS in the time allotted. This highlights the need for unlimited time for deliberate practice to ensure procedural competency. This shift from time-limited to time-unlimited practice is essential to mastery learning. Secondly, learners must be proficiency tested and attain a minimum passing score to complete a SMBL session and achieve mastery. Proficiency testing increases procedural skill retention compared to practice alone.^18^

While deliberate practice and proficiency testing require simulation supplies and significant faculty time, the potential return on investment by medical schools is great if training prevents even a small number of occupational exposures or in-hospital infections. For example, Barsuk et al demonstrated the effectiveness of SBML to reduce central line-associated bacterial infections in an intensive care unit setting and thus reduction in hospital costs.^19^ Additionally, a recent review of SBML studies with Tier 2–Tier 4 research outcomes by Griswold-Theodorson et al highlights the capability of SBML to improve patient care processes and cost reduction, beyond the simulation lab.^20^ Therefore, medical schools should strongly consider using SBML when designing PPE training for medical students.

Criticisms of mastery learning include the time required to implement this teaching modality.^21^ In our study, each SBML session lasted two hours with two faculty and a maximum of 16 students. On average, each student required 15 minutes to complete baseline testing, deliberate practice, and post-testing. However, while 151/153 students completed the entire SBML, two subjects required greater than four post-testing attempts and were still unable to reach mastery due to unanticipated time constraints for additional deliberate practice. These two subjects were pre-clinical students with very limited clinical exposure, early in their second year of medical school, as compared to the other subjects. All students should achieve a predetermined MPS during SBML sessions that do not limit time for deliberate practice. Our sessions were limited to two hours only given institutional constraints, likely contributing to the incomplete performance of the two study subjects. We recommend scheduling SBML sessions with ample time to accommodate all learners and offering additional training sessions for some learners as needed.

## LIMITATIONS

Our findings have several limitations. We conducted this study at a single medical school associated with a university-based teaching hospital, which may limit generalizability. Additionally, the study used a pre-test/post-test design without randomization. Participants received standard hospital PPE training; thus, students’ baseline testing served as the control for the study. Subjects viewed preparatory videos asynchrony-ously, with unclear time between preparation and the intervention; this may have contributed to variations in pre-test scores. While student completion of viewing the assigned videos was not recorded in this study, viewing the videos was a hospital-wide protocol that required attestation. It should be further be noted that we only evaluated students donning and doffing PPE for airborne precautions; other variations in PPE donning and doffing were not evaluated. Scheduling constraints prevented 13 students from fully participating in the study, and two students lacked the deliberate practice time necessary to achieve mastery. Additionally, we conducted this study in a simulated clinical environment. Further studies are required to confirm that PPE donning and doffing skills translate to the clinical environment and to demonstrate skill retention in clinical practice. Lastly, further studies are required to determine the effectiveness of other teaching interventions compared with SBML methodology.

## CONCLUSION

Simulation-based mastery learning improves medical student performance of standard PPE use in a simulated clinical environment. It is an effective instructional method that should be considered by medical schools. Further studies are necessary to demonstrate skill retention in clinical practice.

## Figures and Tables

**Figure:**
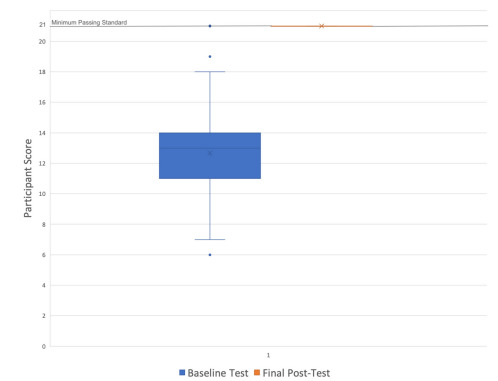
Baseline and final post-test scores of medical students on donning and doffing personal protective equipment, using a 21-item checklist.

**Table:** Demographics of participants.

Characteristic	Students (N = 155)
Male	61 (39.4%)
Female	88 (56.7%)
Other	1 (0.6%)
Declined to answer	5 (3.2%)
POM (preclinical)	101 (65.2%)
EM (clinical)	54 (34.8%)

*POM*, pre-clinical Practice of Medicine course; *EM*, emergency medicine clerkship.
